# Serum Soluble IL-2 Receptors Are Elevated in Febrile Illnesses and Useful for Differentiating Clinically Similar Malignant Lymphomas from Kikuchi Disease: A Cross-Sectional Study

**DOI:** 10.3390/jcm13113248

**Published:** 2024-05-31

**Authors:** Masayuki Fuwa, Yuya Tamai, Ayaka Kato, Motochika Asano, Ichiro Mori, Daichi Watanabe, Hiroyuki Morita

**Affiliations:** 1 Department of General Medicine and Comprehensive Internal Medicine, Gifu University Graduate School of Medicine, Gifu 501-1194, Japan; yuyatamai@gmail.com (Y.T.); kato.ayaka.n4@f.gifu-u.ac.jp (A.K.); asano.motochika.w9@f.gifu-u.ac.jp (M.A.); mori.ichiro.g1@f.gifu-u.ac.jp (I.M.); morita.hiroyuki.d6@f.gifu-u.ac.jp (H.M.); 2 Center for Advanced Medical Care and Clinical Training, Gifu University Hospital, Gifu 501-1194, Japan; watanabe.daichi.k6@f.gifu-u.ac.jp

**Keywords:** soluble IL-2R (sIL-2R), malignant lymphoma (ML), Kikuchi disease

## Abstract

**Background:** The use of serum soluble interleukin 2 receptor (sIL-2R) for the diagnosis of febrile illnesses has not been examined. In this study, febrile patients were classified according to etiology and disease, and serum sIL-2R levels were evaluated. We determined whether serum sIL-2R is a useful marker for differentiating between malignant lymphoma (ML) and non-ML patients and between patients with ML and Kikuchi disease, which present similar clinical manifestations. **Methods:** This study was a cross-sectional study and included 344 patients with uncomplicated hemophagocytic syndrome, who had a fever of 38 °C or higher within 1 week of admission to our institution. Patient serum sIL-2R was measured, and the serum sIL-2R values are shown as median and IQR. **Results:** Serum sIL-2R increased above the upper reference limit in all disease groups with fever. The serum sIL-2R level in ML patients (n = 13) was 4760 (2120–6730) U/mL and significantly higher (*p* < 0.001) than the level of 998 (640–1625) U/mL in non-ML patients (*n* = 331). The serum sIL-2R level in ML patients (*n* = 13) was also significantly higher (*p* < 0.001) compared with that in patients with Kikuchi disease (*n* = 20; 705 (538–1091) U/mL). **Conclusions:** Serum sIL-2R tends to exceed the upper reference limit in patients with febrile illnesses. We conclude that the measurement of serum sIL-2R is useful for differentiating ML from non-ML and ML from Kikuchi disease.

## 1. Introduction

In 1961, Petersdorf and Beeson defined a fever of unknown origin (FUO) as “a fever of 38.3 °C or higher as a sublingual temperature observed several times for more than 3 weeks, with no known cause after hospitalization for more than one week” [1].

In 1991, Durack and Street revised the FUO criteria to “a fever of 38.3 °C of unknown cause that persists for more than 3 weeks and cannot be diagnosed after 3 days of inpatient examination or 3 outpatient examinations” [2]. Diseases that cause fever are so varied that there is no single test that is definitive for differentially diagnosing all of them, which often makes it difficult to have an accurate diagnosis. Infective endocarditis, polymyalgia rheumatica (PMR), adult Still’s disease (AOSD), and malignant lymphoma (ML) are the most common causative diseases of unknown fever [3]. Recently, advances in diagnostic imaging, such as CT and MRI, and histopathological diagnosis using endoscopic ultrasound-guided fine-needle aspiration (EUS-FNA), have made the diagnosis of febrile illnesses easier compared with the past; however, a definitive diagnosis remains difficult to make. It is estimated that up to 50% of cases cannot be definitively diagnosed [3,4,5]. There are many febrile illnesses for which the underlying cause is unclear, such as functional hyperthermia [6], and some diseases have only recently been identified, such as VEXAS syndrome [7].

Recommended diagnostic approaches for febrile illnesses include erythrocyte sedimentation rate, serum C-reactive protein, lactate dehydrogenase, interferon-gamma release assay, rheumatoid factor, heterophile antibody test, serum protein electrophoresis, antinuclear antibody, blood culture, and thoracoabdominal CT [8]. In addition, peripheral blood leukocyte count and procalcitonin are considered tests with a high diagnostic value [9], but they are insufficient for making a definitive diagnosis of febrile illnesses. Therefore, we focused on serum soluble interleukin 2 receptor (sIL-2R) in febrile patients, which has not been adequately evaluated.

Interleukin 2 (IL-2) is an important cytokine that regulates T-cell responses, primarily promoting CD4+ and CD8+ T-cell proliferation. The IL-2 receptor is composed of three subunits [10]. sIL-2R is generated through the proteolytic cleavage of the cell surface receptor (IL-2R). It is released into serum and its concentration is considered a marker of T-cell activation [11]. ML and hemophagocytic syndrome (HPS) [12,13] are representative diseases in which serum sIL-2R levels are elevated in febrile patients; however, in actual clinical practice, high serum sIL-2R levels are caused not necessarily by ML but by other diseases, such as autoimmune and infectious diseases. The measurement of serum sIL-2R is also useful for the evaluation of disease activity in granulomatous diseases, such as sarcoidosis and ML [13]. This is because the activation of various CD4+ T lymphocytes, in addition to the activation of macrophages or dendritic cells, is considered necessary for granuloma formation [14]. As indicated above, serum sIL-2R has long been used for the diagnosis and assessment of activity in certain diseases. Although high serum sIL-2R levels are not disease-specific, they may increase diagnostic certainty when combined with clinical symptoms and imaging studies [15]. However, many physicians are unaware that serum sIL-2R is also elevated in infectious and autoimmune diseases other than ML. Within the scope of our study, we could not find any report that classified febrile patients according to etiologies, such as infections, autoimmune diseases, tumors, Kikuchi disease, drugs, or unknown groups, and discussed their respective serum sIL-2R levels. In our institution, we have many opportunities to treat febrile patients, including patients with unknown fever. The objectives of this study were as follows: (1) to classify febrile patients according to etiology and disease and determine to what extent serum sIL-2R levels are elevated in these patients; (2) to determine whether serum sIL-2R levels differ between febrile ML patients and patients with other febrile diseases (non-ML), as serum sIL-2R has been reported to be elevated in ML [16], and whether this is useful for differentiating ML; and (3) to determine whether serum sIL-2R levels differ between ML and Kikuchi disease, both of which are febrile diseases with lymphadenopathy and exhibit similar clinical features. Lymph node biopsy is often required for the diagnosis of ML and Kikuchi disease, making it difficult to differentiate between the two. Therefore, we determined whether there is a difference in serum sIL-2R between these two diseases and whether it is a useful diagnostic marker to differentiate between them.

## 2. Materials and Methods

### 2.1. Patient Inclusion and Exclusion Criteria

This study was designed as a cross-sectional study, in which 774 patients aged 15 years or older who were admitted to the Department of General Medicine, Gifu University Hospital, between June 2004 and December 2020 were observed to have a fever of 38 °C or higher at least once within one week after admission. These patients were admitted with unknown fever. A total of 351 patients whose serum sIL-2R was measured three days between admission and discharge from the hospital were included in the study. Patient sex, age, clinical diagnosis, and blood test results, including serum sIL-2R, were extracted from the medical records of Gifu University Hospital. The decision to measure serum sIL-2R in febrile patients was made by individual physicians. There were a few patients in whom serum sIL-2R was measured more than once. In those patients, the highest value was used. When measuring serum sIL-2R, it was not considered whether the patient was febrile or not. If a patient had a disease that caused fever before admission, the name of the disease leading to admission was included in the analysis.

First, we analyzed patients who developed HPS because these patients might have elevated serum sIL-2R levels regardless of the cause of their fever. In this study, there were seven patients with HPS complications: two with systemic lupus erythematosus (SLE), two with adult Still’s disease (AOSD), one with granulomatosis polyangiitis (GPA), one with viral infection, and one unknown (Table 1). The serum sIL-2R level in the HPS-complicated group (*n* = 7) was 1620 (1220–5070) U/mL, which was significantly higher than the level of 1058 (646–1700) U/mL in the HPS-uncomplicated group (*n* = 344) (*p* = 0.03) (Supplementary Table S1). Therefore, a total of 344 febrile patients were included in this study, excluding those with HPS. 

### 2.2. Classification of Patients

Patients whose serum sIL-2R was measured were classified into an infectious disease group, an autoimmune disease group, a tumor group, a Kikuchi disease group, a drug fever group, or an unknown group based on the etiology and the final diagnosis. Patients with diseases for which the cause of fever could not be identified were classified into the unknown group. The infectious disease group was further classified as bacterial, viral, fungal, tuberculosis, and rickettsial. Because of the large number of diseases in the autoimmune disease group, diseases with five or more patients were included in the analysis. Autoimmune diseases included SLE, AOSD, Behcet’s disease, microscopic polyangiitis (MPA), giant cell arteritis (GCA), Takayasu’s arteritis (TAK), rheumatoid arthritis (RA), PMR, dermatomyositis (DM)/polymyositis (PM), remitting seronegative symmetrical synovitis with pitting edema (RS3PE) syndrome, pseudogout (CPPD), and TAFRO syndrome (TAFRO). The tumor group was classified into ML and other tumors, with ML being further classified by histology.

### 2.3. Evaluation of Serum sIL-2R 

Patients with fever were classified according to etiology. Infectious and autoimmune diseases were classified according to the disease type, and tumors were classified into ML and other tumors. We also determined whether differences in serum sIL-2R levels occurred between febrile ML and non-ML patients. Furthermore, we examined whether differences in serum sIL-2R occurred between patients with ML and Kikuchi disease. Because ML includes intra-vascular lymphoma (IVL) without superficial lymphadenopathy, we also compared serum sIL-2R levels between ML patients, excluding those with IVL and Kikuchi disease.

### 2.4. Laboratory Examination

Serum sIL-2R was measured using a chemiluminescence immunoassay (CLIA) (Sekisui Medical, Tokyo, Japan, Nanopia IL-2R), and the reference value is 145–519 U/mL.

### 2.5. Statistical Analysis

Continuous variables are expressed as median [IQR]. The Mann–Whitney U test was used to test the difference in serum sIL-2R between men and women, the difference in serum sIL-2R between HPS-complicated and -uncomplicated groups, the difference in serum sIL-2R between ML patients with and without fever, and the difference in serum sIL-2R between patients with ML and Kikuchi disease. *p* < 0.05 was considered significant. The level of serum sIL-2R was evaluated by measuring the area under the receiver operating characteristic (ROC) curve (AUROC), and the cutoff values, sensitivity, and specificity were also analyzed. The cutoff values for the AUROC were defined using the Youden index.

The degree of correlation between patient age and serum sIL-2R was determined using Spearman’s rank correlation coefficient. EZR was used for statistical analysis as this software (version 1.61) extends the functions of R and R Commander. It is freely distributed on the website of Saitama Medical Center, Jichi Medical University [17].

### 2.6. Ethical Approval

The study protocol was reviewed and approved by the Investigational Review Committee of Gifu University Graduate School of Medicine (Approval No. 2021-079, 12 May 2021) and conformed to the provisions of the Declaration of Helsinki. By adopting the opt-out method, we were able to allow patients to decline participation in the disclosure documents.

## 3. Results

### 3.1. Patient Clinical Background

The clinical background of the 344 patients included in this study is listed in Table 1. There were 178 males (51.7%) and 166 females (48.3%), with a median age of 63.5 years (Table 1). Serum sIL-2R levels did not differ between the sexes (*p* = 0.36) (Supplementary Figure S1A) and were not affected by age (R = 0.10, *p* = 0.06) (Supplementary Figure S1B). The composition of each group by etiology was 87 patients (25.2%) in the infectious disease group, 168 (48.8%) in the autoimmune disease group, 35 (10.2%) in the tumor group, 20 (5.8%) in the Kikuchi disease group, 11 (3.2%) in the drug fever group, and 23 (6.7%) in the unknown group (Table 1).

### 3.2. Serum sIL-2R Levels by Etiology and Disease

**Figure 1:**
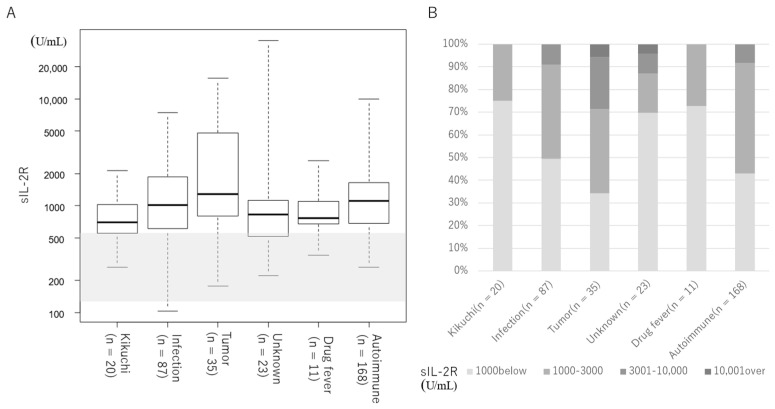
Serum sIL-2R levels by etiology. Shaded areas indicate the reference values for sIL-2R (145 –519 U/mL). (**A**) Median value for each group. The median is indicated by the horizontal bar inside the box. IQR is indicated by the horizontal bars at the top and bottom of the box. Horizontal bars at the top and bottom outside the box indicate the maximum and minimum values. (**B**) Distribution of serum sIL-2R levels by etiology.

**Figure 2:**
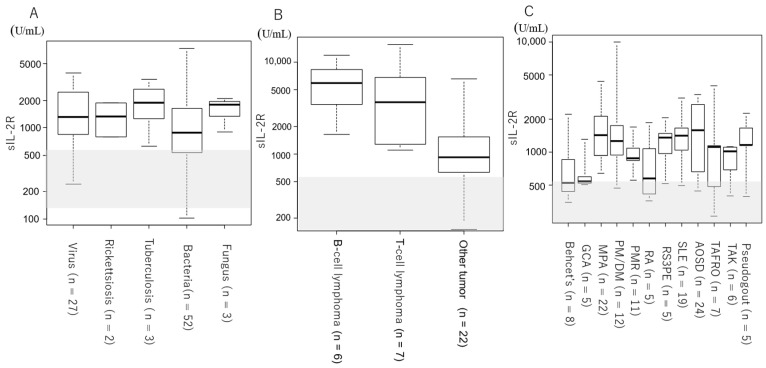
Serum sIL-2R levels by disease. Shaded areas indicate the reference values for sIL-2R (145–519 U/mL). (**A**) Patients with infectious diseases. (**B**) Patients with tumors. (**C**) Patients with autoimmune diseases.

Figure 1 and Table 1 show the serum sIL-2R levels by etiology and disease. For all groups, serum sIL-2R levels were above the upper reference limit (Table 1, Figure 1A). Two cases of ML and one case of unknown origin had serum sIL-2R levels above 10,000 U/mL (Figure 1B). Figure 2 and Table 2 show the serum sIL-2R values by disease. In the infectious disease group (Table 2, Figure 2A), tumor group (Table 2, Figure 2B), and autoimmune disease group (Table 2, Figure 2C), serum sIL-2R levels were above the upper reference limit for all diseases. The serum sIL-2R levels were 5800 (3440–8275) U/mL in B-cell lymphoma (*n* = 7) and 3630 (1590–6230) U/mL in T-cell lymphoma (*n* = 6) (Table 2, Figure 2B).

### 3.3. Comparison of Serum sIL-2R between ML and Non-ML

**Figure 3:**
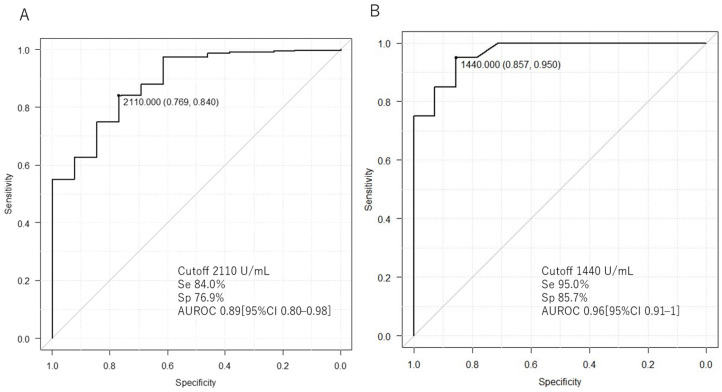
Comparison of serum sIL-2R levels between lymphoma, non-lymphoma, and Kikuchi disease. The area under the curve (AUROC) for the receiver operating characteristic (ROC) analysis of serum sIL-2R is shown. (**A**) Comparison of lymphoma and non-lymphoma. (**B**) Comparison of lymphoma and Kikuchi disease. Se: sensitivity; Sp: specificity.

Figure 3A and Table 3 show a comparison of serum sIL-2R levels between ML and non-ML patients. The serum sIL-2R level of ML patients (*n* = 13) was 4760 (2120–6730) U/mL, which was higher than that of non-ML patients (*n* = 331) at 998 (640–1625) U/mL (*p* < 0.001; Table 3). The AUROC of serum sIL-2R in ML and non-ML patients was 0.89 [95% confidence interval (CI): 0.80–0.98]. The cutoff value to distinguish between the two was 2110 U/mL, with a sensitivity of 84.0% and a specificity of 76.9% (Table 3, Figure 3A). 

### 3.4. Comparison of Serum sIL-2R between ML and Kikuchi Disease

Figure 3B and Table 3 show a comparison of serum sIL-2R levels between patients with ML and Kikuchi disease. The serum sIL-2R level in ML patients (*n* = 13) was 4760 (2120–6730) U/mL, which was higher than the level of 705 (538–1091) U/mL in patients with Kikuchi disease (*n* = 20) (*p* < 0.001; Table 3). The AUROC was 0.96 (95% CI: 0.91–1). The cutoff value to differentiate between the two was 1440 U/mL, with a sensitivity of 95% and a specificity of 85.7% (Table 3, Figure 3B). The serum sIL-2R levels in ML patients, excluding those with IVL and Kikuchi disease, were also measured. The serum sIL-2R level in ML patients, excluding those with IVL (*n* = 10), was 5265 (2222–9167) U/mL, which was higher than the level of 705 (538–1091) U/mL in patients with Kikuchi disease (*n* = 20) (*p* < 0.001; Supplementary Table S2). The cutoff value to differentiate between the two was 1240 U/mL, with a sensitivity of 85.0% and a specificity of 90.0% (Supplementary Figure S1C).

## 4. Discussion

In this study, we showed that serum soluble IL-2 receptor is elevated in patients with febrile diseases in general and is also a useful marker for differentiating between malignant lymphoma and Kikuchi disease, which present similar clinical manifestations. There are two strengths to this study. We first classified febrile patients according to etiology and then further classified them according to disease, and we measured their serum sIL-2R levels. We showed that serum sIL-2R increased above the upper reference limit in febrile patients. Other than febrile illnesses, serum sIL-2R may be elevated in patients with chronic maintenance dialysis [16]; however, our search did not identify any studies showing that it is elevated in other nonfebrile illnesses. It is tempting to consider ML when serum sIL-2R levels are even slightly elevated, but this is incorrect, and it should be noted that serum sIL-2R is frequently higher than the upper reference limit in febrile diseases. Compared with healthy subjects, serum sIL-2R is elevated in patients with T-cell-mediated diseases, such as multiple sclerosis, type 1 diabetes, and RA [18,19,20,21,22]; however, the specific degree to which it is elevated has not yet been examined. Therefore, this study presents a new finding that shows to what extent serum sIL-2R levels are specifically increased in various diseases. In this study, there were three cases in which serum sIL-2R exceeded 10,000 U/mL. Similar to existing reports [23], a marked increase in serum sIL-2R levels in patients with ML was also notable. In one case classified in the unknown group, the serum sIL-2R level was markedly elevated at 35,200 U/mL. This patient was admitted to the hospital because of a fever of unknown origin and multiple enlarged lymph nodes were observed. A lymph node biopsy was scheduled, but a definitive diagnosis could not be made because the patient’s condition deteriorated, leading to death.

Ohno et al. reported that the median serum sIL-2R level in ML patients was 1600 U/mL, whereas patients with B-cell lymphoma exhibited a higher level compared with patients with T-cell lymphoma [23]. The median serum sIL-2R level in patients with B-cell lymphoma was higher in the present study; however, the serum sIL-2R levels of ML patients as a whole were higher compared with Ohno et al.’s study. The reason for this may be that all MLs in this study were the aggressive types, including DLBCL, IVL, and peripheral T-cell lymphoma.

Kanda et al. reported that serum sIL-2R levels were higher in febrile ML patients compared with nonfebrile ML patients [10]. The serum sIL-2R level in febrile patients was 8460 (3545–16,650) U/mL in the ML group (*n* = 48), which was higher than that in the non-ML group (*n* = 373) at 1240 (721–2450) U/mL (*p* < 0.001). In febrile patients, the AUROC, sensitivity, and specificity were 0.88, 81.2%, and 82.3%, respectively, when the cutoff value of serum sIL-2R level to discriminate between the two diseases was defined as 3250 U/mL [10]. The cutoff value of serum sIL-2R for ML and non-ML in this study was 2110 U/mL, which was lower. The reason may be that the median serum sIL-2R level in the Kanda et al. study was higher in febrile ML and non-ML patients compared with that in our study. In addition, Kanda et al. included patients with HPS in the ML and non-ML groups. In the present study, ML accounted for 3.8% of cases in the total number of patients, which was a lower percentage compared with the Kanda et al. study, and all HPS patients were excluded. This may be the reason for the difference in the cutoff value for the serum sIL-2R level between ML and non-ML patients.

The second strength of this study is that we were able to demonstrate that serum sIL-2R is a useful marker for differentiating between ML and Kikuchi disease, which have similar clinical manifestations. Kikuchi disease is a rare benign disease involving fever and lymphadenopathy, which was first reported in young Japanese women. The disease has been reported worldwide irrespective of ethnicity, age, or gender [24]. Kikuchi disease is an acute or subacute disease, often with lymphadenopathy, and symptoms last two to three weeks [25]. Although a histological diagnosis of Kikuchi disease may be necessary to differentiate it from ML, it is not practical to uniformly perform lymph node biopsy, especially considering the complications associated with lymph node biopsies, such as bleeding, wound infection, and scarring. In practice, differentiating ML from Kikuchi disease based on the clinical course is frequently performed, which makes early diagnosis problematic. In the present study, serum sIL-2R levels were significantly higher in patients with ML compared with patients with Kikuchi disease, which suggests that serum sIL-2R may be a useful marker for differentiating these two diseases. Although there are characteristic imaging findings in both diseases [26], there have been no reports of serum markers being useful in differentiating the two. Thus, we consider this to be a new finding.

In ML, CD4+ T-cell stimulation causes high sIL-2R production [27], whereas, in Kikuchi disease, pathologically CD68-positive plasmacytoid monocytes and histiocytes with predominantly CD8-positive T lymphocytes are a hallmark [28]. These differences are likely to result in large differences in serum sIL-2R levels between the two groups.

This study has several limitations. First, the patient population may be biased because inpatients at a single center were selected for the analysis. Therefore, the analysis of patients from multiple institutions, including outpatients, should be considered in future research. Second, our institution often treats febrile patients, who constituted the participants of this study. Therefore, the results may not necessarily apply to patients without fever. Third, when a patient with an existing disease, such as an autoimmune disease, develops a fever because of a secondary disease, such as an infectious disease, we classified the patient according to the febrile disease that caused the admission. Therefore, the preexisting disease may have had a small effect on serum sIL-2R levels. Fourth, cases in which a definitive diagnosis could not be made were classified into the unknown group. Therefore, some cases of ML that could not be diagnosed might have been classified as non-ML.

## 5. Conclusions

In conclusion, serum sIL-2R levels were higher than normal in febrile patients, even in non-ML patients. It may be incorrect to strongly suspect ML only because of slightly elevated serum sIL-2R levels. However, the measurement of serum sIL-2R is useful for differentiating between ML and Kikuchi disease, and a lymph node biopsy may be omitted in cases with low values.

## Figures and Tables

**Table 1:** Background of patients hospitalized with a fever of >38 °C. HPS: hemophagocytic syndrome; IQR: interquartile range.

	Include HPS	Exclude HPS	Age, Years	Women (%)	sIL-2R (U/mL)
Total	351	344	63.5 (39–75)	166 (48)	1058 (646–1700)
Infection	88	87	64 (41–73.50)	31 (36)	1010 (607–1865)
Autoimmune	173	168	62 (38–75)	93 (55)	1105 (687–1643)
Tumor	35	35	73 (62.75–80.00)	16 (46)	1408 (799–5420)
Drug fever	11	11	57 (51.50–75.00)	5 (45)	764 (641–1200)
Kikuchi	20	20	23 (18.75–33.25)	11 (55)	705 (538–1091)
Unknown	24	23	69 (52.00–78.00)	9 (36)	824 (220–35,200)

**Table 2:** Number of patients and serum sIL-2R levels by disease.

	*n*	sIL-2R (U/mL)
***Infection***	87	1010 (607–1865)
bacteria	52	874 (537–1595)
virus	27	1303 (841–2443)
Tuberculosis	3	1870 (1249–2627)
Fungus	3	1780 (1337–1936)
Rickettsiosis	2	1328 (1062–1594)
***Tumor***	35	1408 (799–5420)
**ML**	13	4760 (2120–6730)
**T-cell ML**	6	3630 (1590–6230)
ATLL	1	6730
T-LBL	1	1106
PTCL	3	4730 (3630–10,115)
EATL	1	1277
**B-cell ML**	7	5800 (3440–8275)
DLBCL	4	7890 (4880–10,460)
IVL	3	4760 (3190–5665)
**Other tumor**	22	912 (652–1460)
***Autoimmune***	168	1105 (687–1643)
Behcet’s	8	523 (438–792)
GCA	5	541 (523–595)
MPA	22	1410 (991–2012)
PM/DM	12	1250 (944–1667)
PMR	11	871 (833–1085)
RA	5	573 (416–1070)
RS3PE	5	1340 (973–1480)
SLE	19	1400 (1045–1645)
AOSD	24	1575 (670–2665)
TAFRO	7	1100 (485–1140)
TAK	6	1013 (764–1094)
CPPD	5	1160 (1160–1650)

Note: ML: malignant lymphoma; ATLL: adult T-cell leukemia/lymphoma; T-LBL: precursor T-lymphoblastic lymphoma; PTCL: peripheral T-cell lymphoma; EATL: enteropathy-associated T-cell lymphoma; DLBCL: diffuse large B-cell lymphoma; IVL: intra-vascular lymphoma; Behcet’s: Behcet’s disease; GCA: giant cell arteritis; MPA: microscopic polyangiitis; PM/DM: dermatomyositis (DM)/polymyositis; PMR: polymyalgia rheumatica; RA: rheumatoid arthritis; RS3PE: remitting seronegative symmetrical synovitis with pitting edema; SLE: systemic lupus erythematosus; AOSD: adult Still’s disease; TAFRO: TAFRO syndrome; TAK: Takayasu’s arteritis; CPPD: pseudogout.

**Table 3:** Comparison of serum sIL-2R levels between lymphoma and non-lymphoma, as well as between lymphoma and Kikuchi disease. ML: malignant lymphoma.

	***n***	**sIL-2R (U/mL)**	***p*-Value**
ML	13	4760 (2120–6730)	<0.001
non-ML	331	998 (640–1625)	
	***n***	**Median [25–75%]**	***p*-Value**
ML	13	4760 (2120–6730)	<0.001
Kikuchi	20	705 (538–1091)	

## Data Availability

Data are available upon reasonable request.

## Supplementary Materials

The following supporting information can be downloaded at: https://www.mdpi.com/article/10.3390/jcm13113248/s1, Figure S1. (A) The relationship between serum sIL-2R levels and gender. (B) The relationship between serum sIL-2R levels and age, as determined using Spearman’s rank correlation coefficient. (C) ROC analysis of serum sIL-2R levels in ML patients, excluding those with IVL and Kikuchi disease. Table S1. Comparison of serum sIL-2R between HPS and non-HPS. Table S2. Serum sIL-2R in ML patients, excluding those with IVL and Kikuchi disease.
