# Supplementary material for: Serum Soluble IL-2 Receptors Are Elevated in Febrile Illnesses and Useful for Differentiating Clinically Similar Malignant Lymphomas from Kikuchi Disease: A Cross-Sectional Study
Source: J Clin Med. 2024 May 31;13(11):3248. doi: 10.3390/jcm13113248 (PMC11173182; doi:10.3390/jcm13113248)
Supplement: Supplementary file 1 [file jcm-13-03248-s001.zip › supplement table.pdf]

Supplement Table S1

	n	Median [25%–75%]	*P*-value
HPS	7	1,620 [1,220–5,070]	0.03
non-HPS	344	1,058 [646–1,700]	

Supplement Table S2

	n	Median [25%–75%]	*P*-value
ML(non IVL)	10	5,265 [2,222–9,167]	<0.001
Kikuchi	20	705 [538–1,091]	
