# Supplementary figures and images for: Serum Soluble IL-2 Receptors Are Elevated in Febrile Illnesses and Useful for Differentiating Clinically Similar Malignant Lymphomas from Kikuchi Disease: A Cross-Sectional Study
Source: J Clin Med. 2024 May 31;13(11):3248. doi: 10.3390/jcm13113248 (PMC11173182; doi:10.3390/jcm13113248)

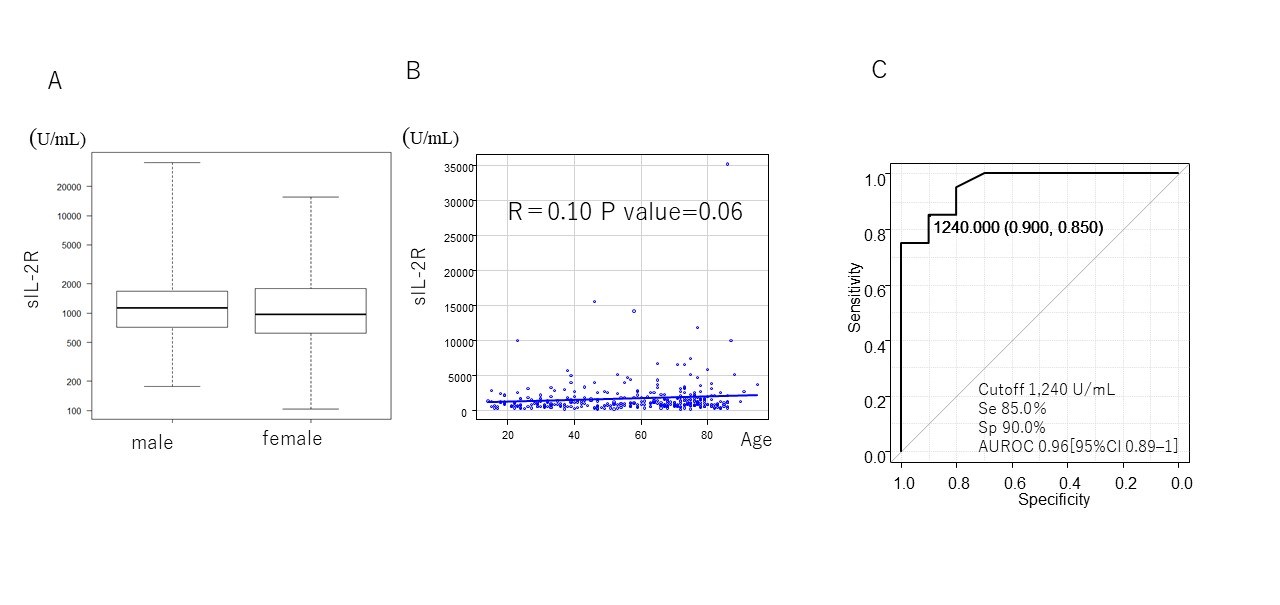

Supplement: Supplementary file 1 [file jcm-13-03248-s001.zip › supplement Figure S1.JPG]
